# Mechanisms of pyrethroid resistance in Culicidae mosquitoes from Hainan Island, China

**DOI:** 10.1186/s13071-025-07052-y

**Published:** 2025-10-14

**Authors:** Qingyun Huang, Yunfei Zhou, Jiabao Xu, guofa zhou, Qiaolu Guo, Yixuan Duan, Guiyu Zheng, Menglou Zhu, Saifeng Zhong, Daibin Zhong, Faxing Fu, Si Wen, Yu Jiang, Tianya He, Suhua Liu, Gang Lu, Yiji Li, Tingting Li

**Affiliations:** 1 Key Laboratory of Tropical Translational Medicine of Ministry of Education, College of basic medical sciences, Hainan Medical University, Haikou, China; 2 Department of Pathogen Biology, College of Basic Medical Sciences, Hainan Medical University, Haikou, China; 3 Hainan Medical University-The University of Hong Kong Joint Laboratory of Tropical Infectious Diseases, Hainan Medical University, Haikou, China; 4 Department of Immunology and Microbiology, School of Basic Medical Sciences, Zhejiang Chinese Medical University, Hangzhou, Zhejiang China; 5 Program in Public Health, College of Health Sciences, University of California at Irvine, Irvine, CA USA; 6 Clinical laboratory, Sanya Central Hospital (Hainan Third Peoples Hospital), Sanya, China

**Keywords:** Mosquitoes, Pyrethroids, *Kdr*, Piperonyl butoxide (PBO), Insecticide resistance

## Abstract

**Background:**

Mosquito-borne diseases represent critical global public health threats. Insecticide-based prevention and interventions remain essential for disease and vector management. However, insecticide resistance in mosquitoes threatens the effectiveness of these management measures. This study investigated the susceptibility to pyrethroid insecticides and associated resistance mechanisms in five dominant mosquito populations on Hainan Island, China.

**Methods:**

World Health Organization (WHO) tube bioassays were conducted to evaluate insecticide resistance profiles in *Aedes albopictus, Culex quinquefasciatus, Armigeres subalbatus, Aedes aegypti, and Culex tritaeniorhynchus*. We assessed the synergistic effects of pre-exposure to 4% piperonyl butoxide (PBO) on deltamethrin mortality rates in *Aedes albopictus* and *Culex quinquefasciatus* populations. We genotyped *kdr* alleles at codon 1534 of the voltage-gated sodium channel gene in *Ae. albopictus* and at codon 1014 in *Cx. quinquefasciatus*.

**Results:**

All five mosquito species exhibited significant resistance to pyrethroid insecticides. *Ae. albopictus* populations from seven localities were resistant to 0.25% permethrin, 0.03% deltamethrin, and 0.03% alpha-cypermethrin, with mortality rates ranging from 0% to 35.0%, 4.0% to 51.0%, and 2.0% to 27.0%, respectively. Similarly, *Cx. quinquefasciatus* populations from five sites demonstrated resistance to 0.25% permethrin, 0.4% deltamethrin, and 0.5% alpha-cypermethrin, with all mortality rates below 90%. Three *Armigeres subalbatus* populations also exhibited resistance to permethrin, deltamethrin, and lambda-cyhalothrin, with one population showing probable resistance to deltamethrin. Pre-exposure to 4% PBO significantly increased mortality rates in both *Ae. albopictus* and *Cx. quinquefasciatus* compared with mosquitoes exposed to deltamethrin alone. However, PBO pre-exposure only partially restored the susceptibility of the mosquitoes to pyrethroids. Molecular analysis revealed a higher frequency of *kdr* mutations (F1534C and F1534S) in resistant *Ae. albopictus* (mean 67.6% ± 24.3%) compared with susceptible mosquitoes (mean 31.6% ± 12.4%) across four of the five populations. For *Cx. quinquefasciatus*, the *kdr* mutation frequency was significantly greater in resistant mosquitoes (mean 82.9% ± 15.0%) than in susceptible mosquitoes (mean 52.8% ± 32.6%).

**Conclusions:**

The results indicated potential multiple resistance mechanisms in mosquitoes in Hainan and highlight the need for systematic monitoring and mapping of insecticide resistance. Innovative mosquito control strategies are needed to support the development and implementation of effective, evidence-based vector control programs.

**Graphical Abstract:**

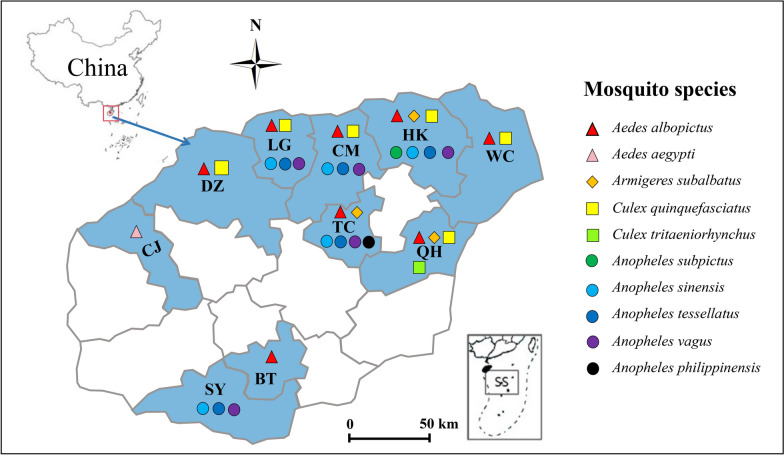

**Supplementary Information:**

The online version contains supplementary material available at 10.1186/s13071-025-07052-y.

## Background

Mosquito-borne diseases, including malaria, dengue fever, Zika, chikungunya, and yellow fever, continue to represent major global public health threats, resulting in hundreds of millions of infections and significant mortality each year [1–3]. These diseases are transmitted by mosquito vectors belonging to genera such as *Anopheles*, *Aedes*, and *Culex* [4, 5]. Rapid urbanization, increasing human mobility, and climate change have collectively facilitated the expansion of these vectors into new geographic regions, thereby increasing the risks and burden of mosquito-borne infections worldwide [6, 7]. The control of mosquito vectors has historically relied on chemical interventions and control measures [8–10]. Among available insecticides, pyrethroids are the most widely used for mosquito vector control globally because of their rapid knockdown effect, relative safety for humans and other animals, and cost-effectiveness [11].

However, the widespread intensive and prolonged use of pyrethroids has led to the development of resistance in mosquitoes globally, especially in regions endemic for mosquito-borne diseases, such as sub-Saharan Africa, South and Southeast Asia, and Latin America [12–14]. *Anopheles* mosquitoes, the primary malaria vectors; *Aedes* mosquitoes, the chief vectors of dengue, Zika, and chikungunya viruses; and *Culex* mosquitoes, the principal vectors of arboviruses (such as Japanese encephalitis virus and West Nile virus), filariasis, and other zoonotic viruses and parasites, have all developed resistance to multiple classes of insecticides [15–19]. This growing threat of mosquito insecticide resistance reduces the efficacy of critical vector control tools and jeopardizes the sustainability of disease prevention programs.

Elucidating the underlying mechanisms of insecticide resistance is essential for developing innovative and sustainable control measures. Target-site mutations and enzyme-mediated metabolic detoxification are among the major mechanisms of resistance to chemical insecticides in mosquitoes [20]. Knockdown resistance (*kdr*), a common form of resistance observed in insects against pyrethroids and DDT, is caused by a mutation in the voltage-gated sodium channel (VGSC) gene, resulting in reduced sensitivity at the target site [21, 22]. To date, *kdr* mutations have been detected in at least 16 species of mosquitoes across the African, Asian, and American continents [23–25]. Metabolic resistance, which involves the breakdown or detoxification of insecticides before they can harm mosquitoes, has been detected in many *Anopheles*, *Aedes*, and *Culex* species and represents one of the primary mechanisms of insecticide resistance, particularly when the *kdr* frequency plateaus [20, 26]. Piperonyl butoxide (PBO), a synergist that enhances insecticide effectiveness, is among the most commonly used diagnostic tools to assess the presence of metabolic resistance [27]. PBO works by inhibiting cytochrome P450 enzymes, which play a key role in metabolic resistance to pyrethroids [28, 29]. Monitoring for *kdr* mutations and metabolic resistance in field mosquitoes is essential for understanding insecticide resistance mechanisms and developing effective strategies to control mosquito-borne diseases.

Hainan Island, the largest island of China in the South China Sea, is located in the tropics and is characterized by high temperatures and abundant precipitation, which creates favorable conditions for the breeding of diverse mosquito species, resulting in a high risk of mosquito-borne disease transmission [30]. A total of 146 species of mosquitoes in 14 genera have been reported on Hainan, including the major disease vectors *Anopheles sinensis*, *Aedes aegypti*, *Aedes albopictus*, and *Culex quinquefasciatus* [30–32]. Historically, mosquito-borne diseases, including malaria, dengue fever, and filariasis, have been prevalent on Hainan Island, China [31]. Dengue fever outbreaks have become more frequent in recent years after approximately 20 years of very low transmission [33–35]. Insecticides, especially pyrethroids, have been widely used for vector control, particularly during disease outbreaks. In addition, insecticides have been widely used for agricultural pest control. In recent years, insecticide resistance among mosquitoes has been reported on Hainan [36, 37]. Given the intensified use of insecticides and the rapid development of insecticide resistance, monitoring the current status of resistance and its underlying mechanisms is imperative. This knowledge is crucial for developing and implementing improved vector control strategies to maintain their effectiveness.

The primary objective of this research was to investigate the pyrethroid resistance profiles of Culicidae mosquitoes on Hainan Island from 2023 to 2024. The second aim was to examine the *kdr* mutation status of different mosquito species. The third aim was to examine the potential role of metabolic resistance by using a synergist bioassay. The results will be useful in determining future vector control strategies.

## Methods

### Study sites and mosquito collection and rearing

**Fig. 1:**
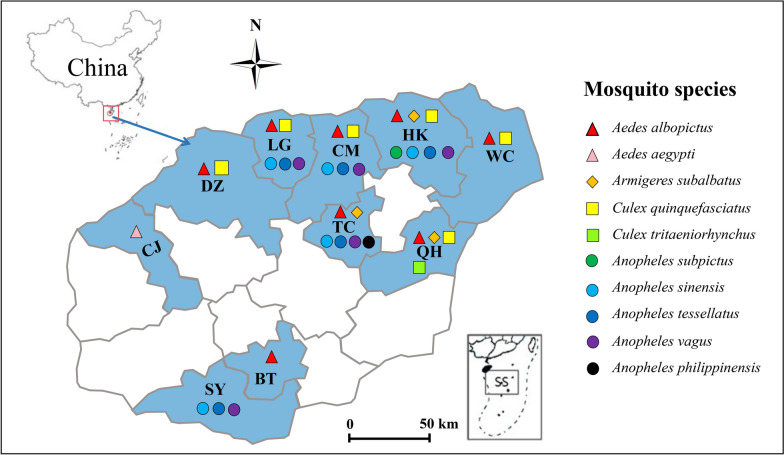
Map of the study sites and mosquito species distribution in Hainan Province, China. HK is Haikou, QH is Qionghai, WC is Wenchang, DZ is Danzhou, TC is Tunchang, BT is Baoting, CM is Chengmai, LG is Lingao, CJ is Changjiang, and SY is Sanya

Mosquito larvae were collected from 10 municipalities and counties on Hainan Island from March 2023 to December 2024 (Fig. 1). Mosquito larvae were collected using the dipping method. Larvae were first identified into *Anopheles*, *Aedes*, *Culex*, *Armigeres*, and other genera. Mosquito species within each genus were identified using PCR methods (see details below) using F0 females after they laid eggs. The eggs of known species were reared to adults for insecticide resistance experiments and follow-up analysis. The locations of the 10 study sites and the mosquito species collected from each site are presented in Fig. 1 and Additional file 1 (Supplementary Table S1). Mosquito collections were systematically conducted across 10 municipalities on Hainan Island. Owing to natural spatial variation in mosquito abundance, only collection sites yielding sufficient samples (≥ 25 individuals per bioassay replicate) were included in insecticide resistance testing.

All the mosquito larvae collected were brought back to the insectary at Hainan Medical University for rearing. Larvae were reared in plastic basins (21 cm × 14 cm × 8 cm) with 0.5–1 L of dechlorinated tap water and fed a 1:4 mixture of yeast powder and fish food. Adult mosquitoes were transferred to mosquito cages (30 cm × 30 cm × 30 cm) and provided with a 10% glucose solution as a food source. The female mosquitoes were blood fed defibrinated goat blood. The larvae were reared in the laboratory until adults emerged. In the laboratory, the temperature was maintained at 28 °C, the relative humidity was 80%, and the light:dark cycle was 16 h:8 h. Nonblood-fed F0–F2 generation female mosquitoes aged 3–5 days were used for the World Health Organization (WHO) tube bioassays.

### Mosquito species identification

For adult mosquito species identification, the mosquitoes were first morphologically divided into *Anopheles, Culex*, *Aedes*, *Armigeres*, and other genera, mainly on the basis of their head, thorax, and abdomen [38]. DNA was subsequently extracted for molecular identification to determine the species. Before DNA extraction, each mosquito sample was surface sterilized with 75% ethanol three times and then placed on filter paper to absorb the alcohol from the surface of the mosquito. Genomic DNA was extracted from two legs of each mosquito individually [39]. Afterwards, the extracted DNA was stored at −20 °C or used immediately for PCR.

Molecular identification of *An. sinensis* was conducted using the species-specific PCR primers *An. sinensis*-F (5ʹ-TGTGAACTGCAGGACACATGAA-3ʹ) and *An. sinensis*-R (5ʹ-AGGGTCAAGGCATACAGAAGGC-3ʹ) [40] (Additional file 2: Supplementary Table S2). PCR amplification was performed in a 25 μL reaction volume with 12.5 μL of PCR Taq Master Mix (2 ×) (Tiangen, China), 1 μL each of the forward and reverse primers at 10 μmol/L, 1 μL of template DNA, and sufficient nuclease-free water to constitute 25 μL. The cycling parameters used included one cycle of denaturation at 95 °C for 5 min, followed by 35 cycles of amplification at 95 °C for 30 s, 52 °C for 30 s, and 72 °C for 1 min, with a final extension at 72 °C for 5 min. PCR products (5 μL) was run on a 1.5% agarose gel with a DL2000 DNA marker (Takara Bio Inc., Shiga, Japan) to confirm the PCR amplification. Examples of agarose gel electrophoresis banding patterns are shown in Additional file 3 (Supplementary Fig. S1).

For the identification of other mosquito species, Sanger sequencing was performed to target a fragment of the mitochondrial cytochrome c oxidase subunit I (*coxI*) gene using the primers LCO1490-F (5ʹ-GGTCAAATCATAAAGATATTGG-3ʹ) and HC02198-R (5ʹ-TAAACTTCAGGGTGACCAAAAAATCA-3ʹ) [41]. The cycling parameters used included one cycle of denaturation at 94 °C for 5 min, followed by 35 cycles of amplification at 95 °C for 30 s, 55 °C for 45 s, and 72 °C for 1 min, with a final extension at 72 °C for 10 min. Sanger sequencing of the PCR-positive products was performed to determine their species.

### Insecticide resistance bioassays

#### Adult resistance bioassays

F1‒F2 females reared from field-collected mosquito larvae were subjected to insecticide susceptibility tests against pyrethroids using the standard WHO insecticide susceptibility tube test [42–44]. For each species of mosquito and each insecticide, four replicates of 25 3–5-day-old nonblood-fed female mosquitoes were subjected to the WHO tube bioassays. In total, we determined pyrethroid insecticide resistance in *Ae. albopictus* at seven collection sites, *Cx. quinquefasciatus* at five collection sites, *Ar. subalbatus* at three sites, and *Ae. aegypti* and *Cx. tritaeniorhynchus* at one site. The concentrations of the tested insecticides included 0.25% permethrin, 0.03% deltamethrin, and 0.03% alpha-cypermethrin for *Ae. albopictus* and *Ae. aegypti* [42]; 0.25% permethrin, 0.4% deltamethrin, and 0.5% alpha-cypermethrin for *Cx. quinquefasciatus* [43]; 0.25% permethrin, 0.05% deltamethrin, and 0.025% lambda-cyhalothrin for *Ar. subalbatus*; and 0.25% permethrin, 0.05% deltamethrin, and 0.5% alpha-cypermethrin for *Cx. tritaeniorhynchus* [43, 44]. The number of knockdown females was recorded every 10 min during the 1 h exposure period. Mortality was scored after the 24 h recovery period. After the bioassay, both the surviving and dead mosquitoes were stored individually in anhydrous ethanol at −20 °C for subsequent DNA analysis.

### Resistance intensity and synergistic effect of piperonyl butoxide

To assess mosquito resistance intensity, WHO bioassays using five- and tenfold concentrations of deltamethrin (a representative pyrethroid insecticide) were employed against the two dominant mosquito populations, *Ae. albopictus* and *Cx. quinquefasciatus*, mosquitoes collected from five sites, i.e., Haikou, Qionghai, Danzhou, Chengmai, and Lingao. *Aedes albopictus* were tested against deltamethrin at concentrations of 0.03%, 0.15%, and 0.3%. Similarly, *Cx. quinquefasciatus* were evaluated for resistance to 0.4%, 2%, and 4% deltamethrin. Nonblood-fed, 3–5-day-old female mosquitoes were exposed to the test papers for 1 h, the number of knockdown individuals was recorded every 10 min, and the mortality rate was determined at 24 h postexposure. There were four replicates for each insecticide, with 25 mosquitoes per replicate.

Synergist assays with piperonyl butoxide (PBO) were performed together with the same mosquito populations and deltamethrin concentrations as described above. The mosquitoes were exposed to 4% PBO test paper for 1 h, followed by exposure to insecticide-treated test papers for 1 h. The tests were conducted with four replicates for each concentration of insecticide and each species of mosquito, with 25 females per replicate, and the mortality rate was determined at 24 h postexposure.

### Molecular identification of *kdr* mutation

Genomic DNA was extracted to detect mutations in pyrethroid insecticide resistance target genes from approximately 30 phenotypically resistant (surviving) and 30 phenotypically susceptible individuals (dead) from each of five sites for *Ae. albopictus* and from each of three sites for *Cx. quinquefasciatus*. These mosquitoes were tested using the standard WHO tube test, and only sites with sufficient surviving and dead samples for molecular *kdr* analysis were included [42, 44]. To identify *kdr* alleles, partial sequences of the voltage-gated sodium channel (VGSC) gene were amplified using the primers *Ae. albopictus-kdr-*F (5ʹ-GAGAACTCGCCGATGAACTT-3ʹ) and *Ae. albopictus-kdr-*R (5ʹ-TAGCTTTCAGCGGCTTCTTC-3ʹ) [45], *Culex-kdr-*F (5ʹ-GTGGAACTTCACCGACTTC-3ʹ) and *Culex-kdr-*R (5ʹ-GCAAGGCTAAGAAAAGGTTAAG-3ʹ) [46], *An-kdr-*F (5ʹ-GACCATGATCTGCCAAGATGGAAT-3ʹ) and *An-kdr-*R (5ʹ-GAGGATGAACCGAAATTGGAC-3ʹ) [47]. The cycling parameters used were the same as those used for the *coxI* primers.

A total of 271 *Ae. albopictus* samples were sequenced for the *kdr* gene, specifically targeting codon 1534, which is associated with pyrethroid resistance in this species. Similarly, a total of 147 *Cx. quinquefasciatus* samples were sequenced for the *kdr* gene to examine mutations at codon 1014. In addition, 92 field adult *Anopheles* mosquitoes (*An. sinensis*, *An. tessellatus*, *An. vagus*, *An. philippinensis*, and *An. subpictus*) from five sites were examined for the L1014F mutation, which is the most common *kdr* variant associated with pyrethroid resistance in anopheline mosquitoes.

### Statistical analysis

For adult bioassays, the phenotypic resistance status was classified according to WHO criteria [42, 44]: populations with mortality < 90% were considered resistant, those with mortality between 90% and 98% were classified as probably resistant, and those with mortality > 98% were classified as susceptible. Individual mosquitoes were categorized as phenotypically susceptible if they died within the 24 h recovery period and as phenotypically resistant if they survived. The association between *kdr* genotypes and resistance phenotypes was assessed using Fisher’s exact test or the *χ*^2^ test (when all *n* > 5), and odds ratios (ORs) with 95% confidence intervals were calculated to quantify the strength of association for each mutation. Differences in mortality rates between the deltamethrin alone and deltamethrin + PBO treatments across the study sites were analyzed using Studentʹs *t* test. One-way analysis of variance (ANOVA) followed by Tukey’s honestly significant difference (HSD) post hoc test was employed to compare mortality rates among the standard, 5× , and 10× concentrations of deltamethrin.

## Results

### Widespread pyrethroid resistance was detected in Culicidae mosquitoes from Hainan Island

Our comprehensive surveillance revealed widespread pyrethroid resistance across all five dominant mosquito species on Hainan Island. The resistance profiles varied by species and geographical location.

**Fig. 2:**
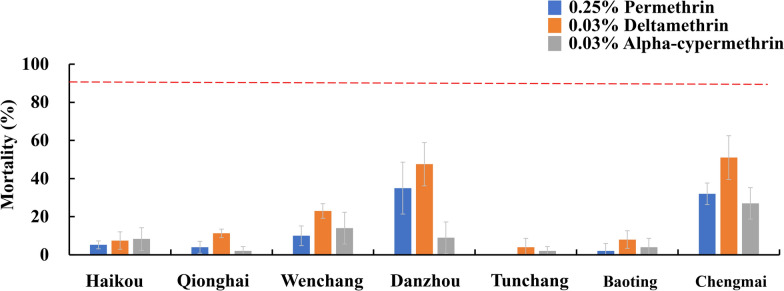
Pyrethroid resistance status of *Aedes albopictus* populations in Hainan. If the mortality is less than 90%, then the population is considered resistant according to the WHO criteria. The error bars represent the standard deviation (std.)

*Aedes albopictus* populations from all seven collection sites (Haikou, Qionghai, Wenchang, Danzhou, Tunchang, Baoting, and Chengmai) exhibited resistance to all three tested pyrethroid insecticides according to the WHO criteria (mortality < 90%) (Fig. 2). The average mortality rates in response to 0.25% permethrin, 0.03% deltamethrin, and 0.03% alpha-cypermethrin were low, ranging from 0% to 35.0% ± 13.6%, 4.0% ± 4.6% to 51.0% ± 11.5%, and 2.0% ± 2.3% to 27.0% ± 8.2%, respectively (Fig. 2). Notably, the mortality rates of the *Ae. albopictus* population from Tunchang were the lowest, with no mortality against permethrin, 4.0% ± 4.6% mortality to deltamethrin, and 2.0% ± 2.3% mortality to alpha-cypermethrin, indicating high-level resistance to all the tested pyrethroids.

Similarly, *Ae. aegypti* collected from Changjiang demonstrated strong resistance to all three pyrethroids, with mortality rates of 0%, 45.0% ± 6.8%, and 10.0% ± 5.2% against permethrin, deltamethrin, and alpha-cypermethrin, respectively (Additional file 2: Supplementary Fig. S1).

**Fig. 3:**
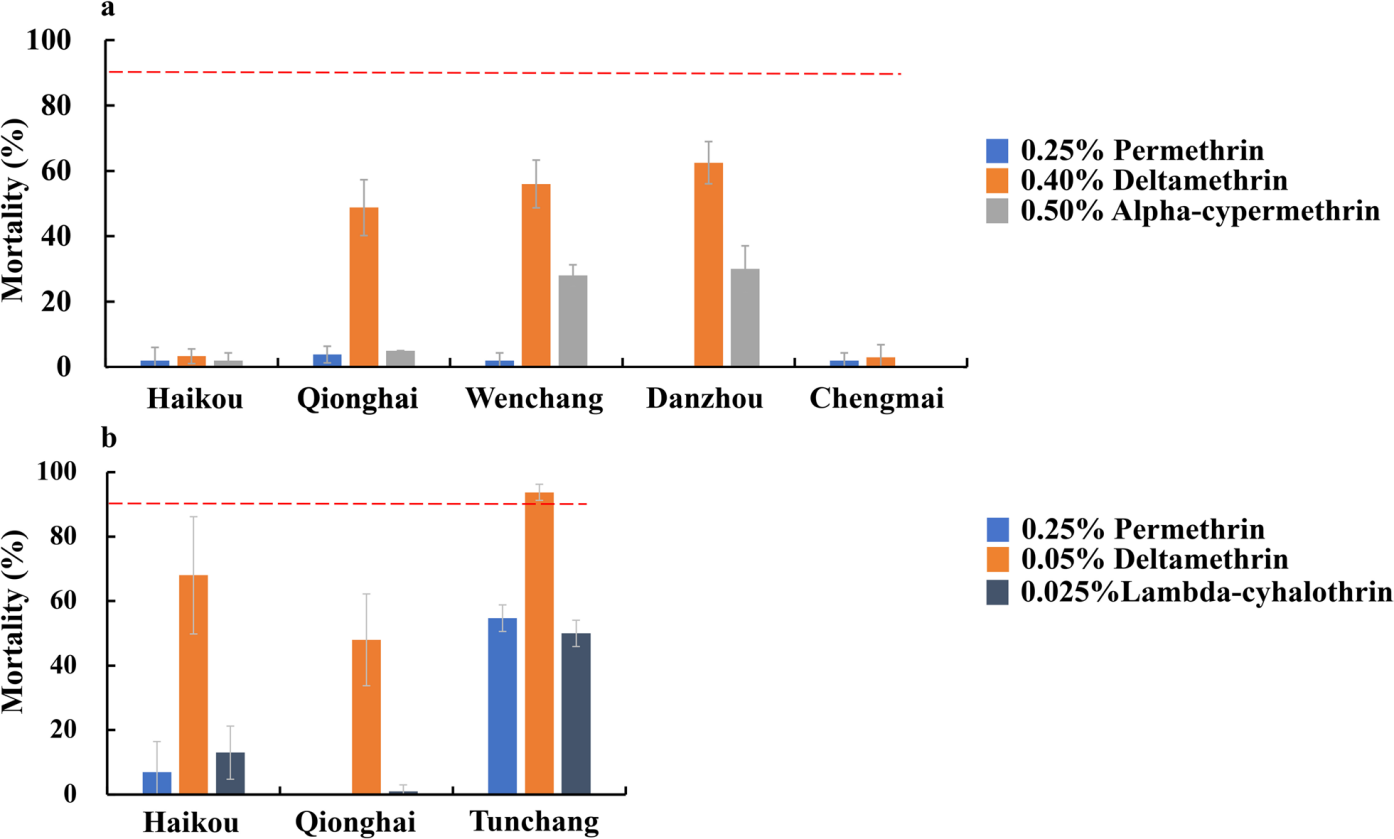
Pyrthroid resistance status of *Culex quinquefasciatus* populations **a** and *Armigeres subalbatus* populations **b** in Hainan. If the mortality is less than 90%, then the population is considered resistant according to the World Health Organization (WHO) criteria. The error bars represent the standard deviation (std.)

*Culex quinquefasciatus* populations from all five sampled locations (Haikou, Qionghai, Wenchang, Danzhou, and Chengmai) also exhibited strong resistance to the tested pyrethroids (Fig. 3a). Exposure to 0.25% permethrin, 0.4% deltamethrin, and 0.5% alpha-cypermethrin resulted in mortality rates of only 0% to 3.8% ± 2.5%, 3.0% ± 3.8% to 62.5% ± 6.5%, and 0% to 30.0% ± 7.1%, respectively. The lowest mortality rates were observed in the Danzhou population for permethrin (0% mortality) and in the Chengmai population for both deltamethrin and alpha-cypermethrin (3.0% ± 3.8% and 0% mortality, respectively).

*Armigeres subalbatus* populations from three sites (Haikou, Qionghai, and Tunchang) were also resistant to multiple pyrethroids (Fig. 3b). When the mosquitoes were exposed to 0.25% permethrin, 0.05% deltamethrin, and 0.025% lambda-cyhalothrin, the mortality rates ranged from 0% to 54.7% ± 4.1%, 48.0% ± 14.2% to 93.8% ± 2.5%, and 1.0% ± 2.0% to 50.0% ± 4.1%, respectively. The Qionghai population exhibited the lowest mortality rates for all three insecticides, with mortality rates of 0%, 48.0% ± 14.2%, and 1.0% ± 2.0%, respectively. The Tunchang population showed probable resistance to deltamethrin, with a mortality rate of 93.8% ± 2.5%.

*Culex tritaeniorhynchus* collected from Qionghai exhibited resistance to all three tested pyrethroids (Additional file 4: Supplementary Fig. S2). The mortality rates were only 2.0% ± 2.3%, 4.0% ± 0%, and 20.0% ± 3.3% for 0.25% permethrin, 0.05% deltamethrin, and 0.5% alpha-cypermethrin, respectively.

### Resistance intensity in *Aedes albopictus* and *Culex quinquefasciatus*

**Fig. 4:**
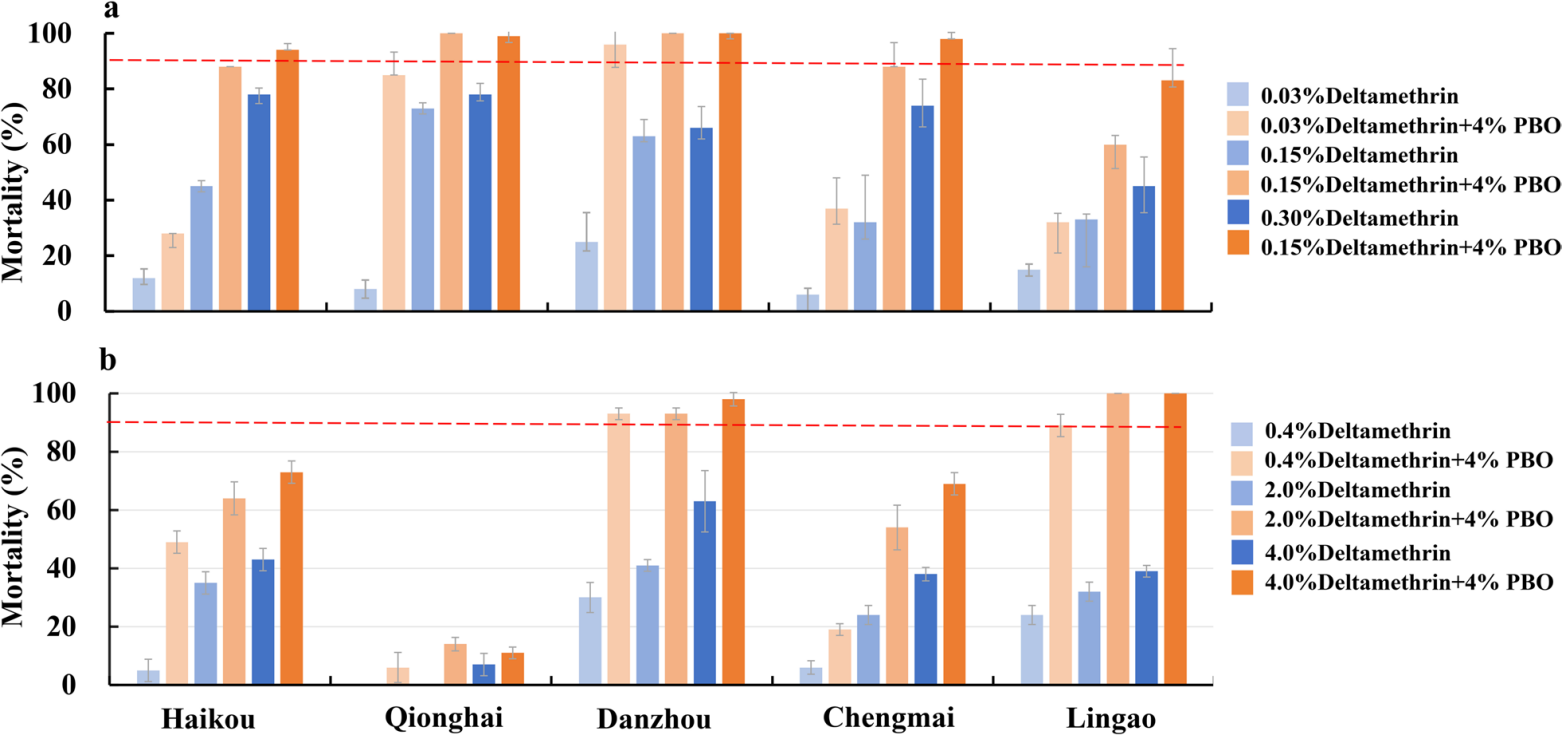
Dose‒response relationships of *Aedes albopictus* populations. **a** and *Culex quinquefasciatus* populations **b** in Hainan with respect to deltamethrin and synergistic effects with piperonyl butoxide (PBO). If the mortality is less than 90%, then the population is considered resistant according to the WHO criteria. The error bars represent the standard deviation (std.)

With respect to *Ae. albopictus*, mortality rates increased significantly when it was exposed to high concentrations of deltamethrin (ANOVA, *F*_(2,26)_ = 605.3, *P* < 0.0001; Tukeyʹs HSD, *P* < 0.0001). When exposed to the standard dose (0.03%), 5× dose (0.15%), and 10× dose (0.30%) of deltamethrin, mortality rates increased from 12.0% ± 3.3% to 78.0% ± 2.3% in Haikou, 8.0% ± 3.3% to 78.0% ± 4.0% in Qionghai, 25.0% ± 10.5% to 66.0% ± 7.7% in Danzhou, 6.0% ± 2.3% to 74.0% ± 9.5% in Chengmai, and 15.0% ± 2.0% to 45.0% ± 10.5% in Lingao (Fig. 4a). Notably, even at the 10× concentration, mortality rates remained below 90% for all the populations, indicating high-intensity resistance to deltamethrin across all the tested sites. Pre-exposure to the 4% PBO synergist significantly increased the mortality rates following deltamethrin exposure in *Ae. albopictus* across all concentrations and locations (*t* test, all *P* < 0.05). The synergistic effect of PBO was particularly pronounced to the standard dose (0.03%), with mortality rates increasing to 28.0% ± 0% to 96.0% ± 5.7% compared with 6.0% ± 2.3% to 25.0% ± 10.5% without PBO. At higher deltamethrin concentrations, the addition of PBO further increased mortality rates, reaching 60.0% ± 3.3% to 100% at the 5× dose and 83.0% ± 11.5% to 100% at the 10× dose for most populations (Fig. 4a).

Similarly, *Cx. quinquefasciatus* populations exhibited dose-dependent responses to deltamethrin, with mortality rates increasing significantly at higher concentrations (ANOVA, *F*_(2, 26)_ = 605.3, *P* < 0.001; Tukeyʹs HSD, *P* < 0.001). When exposed to the standard dose (0.4%), 5× dose (2.0%), and 10× dose (4.0%) of deltamethrin, mortality rates increased from 5.0% ± 3.8% to 43.0% ± 3.8% in Haikou, 0% to 7.0% ± 3.8% in Qionghai, 30.0% ± 5.2% to 63.0% ± 10.5% in Danzhou, 6.0% ± 2.3% to 38.0% ± 2.3% in Chengmai, and 24.0% ± 3.3% to 39.0% ± 2.0% in Lingao (Fig. 4b). The addition of PBO significantly increased the efficacy of deltamethrin against *Cx. quinquefasciatus* (*t* test, all *P* < 0.05), with mortality rates increasing to 6.0% ± 5.2% to 93.0% ± 2.0% at the standard dose (0.4%), 14.0% ± 2.3% to 100% ± 0% at the 5× dose (2.0%), and 11.0% ± 2.0% to 100% ± 0% at the 10× dose (4.0%). The exception was the Qionghai population, whose mortality increased only modestly (6.0% ± 5.2% to 14.0% ± 2.3%), even with pre-exposure to PBO (Fig. 4b).

### *Kdr* mutations were associated with phenotypic resistance in *Aedes albopictus* and *Culex quinquefasciatus*

A total of 271 *Ae. albopictus* mosquitoes (158 phenotypically resistant and 113 phenotypically susceptible) were examined for *kdr* mutations. We detected F1534C and F1534S mutations, with the total *kdr* mutation frequency significantly greater in resistant mosquitoes (mean 67.6% ± 24.3%, range 39.7–83.8%) compared with susceptible mosquitoes (mean 31.6% ± 12.4%, range 17.4–40.0%) across four of the five populations (*χ*^2^ test, all *P* < 0.05; Table 1). The F1534C mutation was significantly associated with phenotypic resistance in most populations, with odds ratios (ORs) of 10.4 (95% CI 3.4–31.5) in Qionghai, 8.5 (95% CI 3.2–22.4) in Chengmai, 4.2 (95% CI 1.8–9.8) in Wenchang, and 3.3 (95% CI 1.2–9.1) in Danzhou, whereas no significant association was detected in Haikou (OR = 1.1, 95% CI 0.3–3.5). Similarly, the F1534S mutation was significantly associated with resistance in Chengmai (OR = 4.5, 95% CI 1.7–12.3) and Qionghai (OR = 5.1, 95% CI 1.6–16.0) but not in Haikou (OR = 0.3, 95% CI 0.1–1.1) or Danzhou (OR = 2.7, 95% CI 0.5–15.0). Neither F1534L nor F1534I mutations were detected at any study site.

**Table 1:** *Kdr* mutation frequency relationship to pyrethroid resistance in different *Aedes albopictus* populations

Study site	Phenotype	Sample size	Allele frequency (%)			Odds ratio (95% CI)	
			Wild-type	F1534C	F1534S	F1534C	F1534S
Haikou	Resistant	30	75.0	18.3	6.7	1.1 (0.3–3.5)^NS^	0.3 (0.1–1.1)^NS^
	Susceptible	17	64.7	14.7	20.6		
Qionghai	Resistant	40	16.3	56.3	27.5	10.4 (3.4–31.5)***	5.1 (1.6–16.0)**
	Susceptible	15	60.0	20.0	20.0		
Chengmai	Resistant	29	20.7	50.0	29.3	8.5 (3.2–22.4)***	4.5 (1.7–12.3)**
	Susceptible	28	62.5	17.9	19.6		
Wenchang	Resistant	30	33.3	38.3	28.3	4.2 (1.8–9.8)**	NA
	Susceptible	30	78.3	21.7	0.0		
Danzhou	Resistant	29	60.3	31.0	8.6	3.3 (1.2–9.1)*	2.7 (0.5–15.0)^NS^
	Susceptible	23	82.6	13.0	4.3		

*NS* no significance, *P < 0.05, ** *P* < 0.01, *** *P* < 0.001

For *Cx. quinquefasciatus,* we analyzed 147 mosquitoes (75 phenotypically resistant and 72 phenotypically susceptible) for *kdr* mutations and identified L1014F and L1014S variants. The L1014F mutation frequency was significantly greater in phenotypically resistant mosquitoes from Wenchang (67.2%) and Danzhou (66.7%) than in phenotypically susceptible mosquitoes (23.9% and 35.7%, respectively) (*χ*^2^ test, *P* < 0.05; Table 2). The odds ratio for the L1014F mutation was 7.8 (95% CI 3.2–19.0) in Wenchang and 4.2 (95% CI 1.7–10.5) in Danzhou, indicating a strong association with pyrethroid resistance. In Qionghai, all resistant mosquitoes carried the L1014F mutation (100%), while susceptible mosquitoes also had a high frequency (83.3%), resulting in an undefined odds ratio. The L1014S mutation was not significantly associated with the resistance phenotype in any of the three populations tested.

**Table 2:** *Kdr* mutation frequency relationship to pyrethroid resistance in different *Culex quinquefasciatus* populations

			Allele frequency (%)			Odds ratio (95% CI)	
Study site	Phenotype	Sample size	Wild-type	L1014F	L1014S	L1014F	L1014S
Wenchang	Resistant	29	27.6	67.2	5.2	7.8 (3.2–19.0)***	NA
	Susceptible	23	76.1	23.9	0.0		
Qionghai	Resistant	25	0.0	100.0	0.0	NA	NA
	Susceptible	21	11.9	83.3	4.8		
Danzhou	Resistant	21	23.8	66.7	9.5	4.2 (1.7–10.5)**	2.0 (0.5–8.6)^NS^
	Susceptible	28	53.6	35.7	10.7		

*NS* no significance, ** *P* < 0.01, *** *P* < 0.001.

### *Kdr* analysis for *Anopheles* mosquitoes was not conducted because of insufficient sample sizes

A total of 92 adult *Anopheles* mosquitoes including 48 *An. sinensis* (52.2%), 23 *An. tessellatus* (25.0%), 11 *An. vagus* (12.0%), 8 *An. philippinensis* (8.7%), and 2 *An. subpictus* (2.2%) were collected from five sampling sites in Hainan. Owing to insufficient numbers of specimens for WHO bioassay testing, a *kdr* analysis for *Anopheles* mosquitoes was not conducted.

## Discussion

Culicidae mosquitoes are widely distributed and capable of transmitting various pathogens, posing a significant threat to public health worldwide, including in China. Malaria was eliminated from Hainan Island in 2019; however, dengue fever has returned to Hainan recently, with several outbreaks [35]. To control outbreaks and prevent potential dengue transmission, insecticides have been widely and frequently applied on the island. Updated information on mosquito insecticide resistance levels on the island is essential for planning and implementing future disease prevention measures. This study presents a comprehensive examination of resistance profiles in multiple mosquito species across Hainan Island, providing a critical reference for the development of integrated control strategies. Our study revealed that major mosquito species (*Ae. albopictus*, *Cx. quinquefasciatus*, *Ar. subalbatus*, and *Ae. aegypti*) in Hainan have developed high-level resistance to pyrethroid insecticides, potentially posing significant challenges for mosquito-borne disease prevention and control programs.

Previous studies have documented the emergence of insecticide resistance in *Ae. albopictus*, *An. sinensis*, and *Cx. quinquefasciatus* populations in Hainan, China [36, 48, 49]. A previous study conducted in 2017–2018 revealed that the mortality rates of *Ae. albopictus* populations ranged from 70% to 96% in response to deltamethrin at different sites on Hainan Island, whereas this study revealed significantly lower mortality rates 6 years later, i.e., with mortality rates ranging from 7% to 51%. The results from nearly all the tests revealed very low mortality across all the mosquitoes and all the insecticides except in *Ar. subalbatus* populations tested against deltamethrin; however, only one population showed probable resistance (with a mortality rate of 90–97%), indicating widespread resistance across species and sites to pyrethroid insecticides. In fact, even at 5× and 10× higher insecticide concentrations, both *Ae. albopictus* and *Cx. quinquefasciatus* showed mortality rates less than 90%, indicating high-intensity resistance. Pyrethroid insecticides have been the primary chemical control method for mosquito management in China over the past three decades, leading to widespread resistance development in most of the surveyed regions [50]. Local insecticide usage surveys on Hainan Island have documented frequent pyrethroid applications for both indoor and outdoor vector control programs [36]. The deltamethrin resistance observed in our field populations is consistent with this sustained pyrethroid selection pressure and reflects the broader resistance patterns documented throughout China.

The synergist PBO has been commonly used to determine metabolic resistance, especially resistance mediated by cytochrome P450 enzymes [51]. A previous 2017–2018 study revealed that PBO exposure resulted in 100% mortality in *Ae. albopictus* when exposed to a standard dose of deltamethrin [36]. However, the results from the current study revealed that even after PBO exposure, *Ae. albopictus* populations exhibited mortality rates below 30% against the standard concentration of deltamethrin, indicating significantly elevated resistance levels in this species. These results concur with the results of a previous study conducted in Guangzhou, Guangdong Province, China [45] indicating that *Ae. albopictus* can develop resistance rapidly. The results of the PBO synergist assay also suggest the involvement of other metabolic resistance mechanisms, such as glutathione S-transferase (GST) enzyme activities, because PBO exposure mainly inhibits P450 enzyme activity, and mortality did not reach 100% after PBO exposure. These findings further suggest the involvement of additional resistance mechanisms in *Ae. albopictus*, potentially indicating the involvement of multiple resistance mechanisms, as the 2017–2018 study revealed 100% mortality in *Ae. albopictus* against deltamethrin after PBO exposure. Similarly, *Cx. quinquefasciatus* also exhibited strong resistance to deltamethrin, potentially through multiple resistance mechanisms, which is consistent with findings reported from several countries including Cameroon, Brazil, India, Saudi Arabia, and the USA [52–56].

The high levels of pyrethroid resistance documented in *Ae. albopictus* and *Cx. quinquefasciatus* throughout Hainan may present challenges for controlling associated vector-borne diseases, particularly dengue fever, under field conditions. However, our findings on the effectiveness of PBO in significantly increasing mosquito mortality demonstrate promising potential for field applications. Insecticide-treated nets (ITNs) combining pyrethroids with PBO represent an innovative vector control tool initially developed for malaria management. These nets received a conditional endorsement from the World Health Organization (WHO) in 2017 [57], with field studies demonstrating their effectiveness in reducing malaria incidence compared with pyrethroid-only ITNs [58]. Multiple studies have confirmed that compared with standard long-lasting insecticidal nets, pyrethroid-PBO combinations (incorporating deltamethrin, permethrin, and alpha-cypermethrin) induce significantly higher mortality in *Anopheles* mosquitoes in both field and experimental hut settings [59–61]. Despite these promising results, the application of PBO in insecticide formulations targeting other vector-borne diseases, such as dengue fever, remains relatively limited. On the basis of our findings, we recommend the strategic evaluation and potential incorporation of PBO in field control programs targeting mosquito vectors in Hainan and comparable regions to enhance insecticidal efficacy against increasingly resistant populations.

*Armigeres subalbatus* (Coquillett, 1898), a common yet often neglected species, has attracted increasing attention because of its potential role as a vector for Zika virus and other pathogens. *Armigeres subalbatus* is widely distributed throughout southern China, primarily south of the Yangtze River [62], and has been shown to transmit several pathogens, including filariasis and Japanese encephalitis virus [63–65]. Our previous study confirmed the widespread distribution of *Ar. subalbatus* throughout Hainan Island [32]. The results of the current investigation reveal that *Ar. subalbatus* populations from three sites in Hainan have developed resistance to pyrethroid insecticides, which raises additional concerns regarding comprehensive vector control. Currently, there is limited global research on insecticide resistance in this species. The relationship between pyrethroid resistance and specific *kdr* mutations in *Ar. subalbatus* remains unexplored. Further investigations are needed to elucidate the genetic and biochemical mechanisms underlying insecticide resistance in this increasingly important vector species.

This study has several limitations. One limitation is that we did not conduct insecticide resistance tests with *Anopheles* mosquitoes because of the limited number of samples collected. However, a previous study reported high pyrethroid insecticide resistance levels in *An. sinensis* populations on Hainan Island [37, 40]. The relationship between the phenotypic resistance to pyrethroid insecticides in *Anopheles* populations from Hainan and *kdr* mutations requires further study. Although we tested the impact of PBO exposure on mosquito mortality, other synergists, such as S,S,S-tributyl phosphorotrithioate (DEF, a carboxylesterase inhibitor) [66, 67] or diethyl maleate (DEM, a glutathione S-transferase inhibitor) [68, 69], could be used to confirm the existence of AChE- and GST-mediated resistance mechanisms. In addition, direct enzymatic assays to measure metabolic enzyme activities before and after insecticide exposure could provide more definitive evidence and validation of metabolic resistance mechanisms [51, 62]. The evaluation of resistance to other classes of insecticides commonly used for agricultural and public health activities is also important for integrated vector management. Furthermore, this study was conducted under controlled laboratory conditions using standardized WHO bioassays, and the field effectiveness of current control interventions may differ because of factors such as mosquito behavior, environmental conditions, and insecticide application methods. The cross-sectional nature of our survey means that it provides current resistance status but cannot be used to predict temporal trends or resistance development patterns. Future studies should evaluate the operational performance of vector control tools against these resistant populations under field conditions.

## Conclusions

The results demonstrate widespread pyrethroid resistance in mosquito populations across Hainan Island, which may significantly impact current vector control strategies. Conventional pyrethroid-based approaches may be becoming less effective under laboratory conditions, underscoring the urgent need for alternative control approaches. Integrated vector management (IVM) strategies combining chemical, biological, and environmental control measures should be considered to mitigate the impacts of mosquito resistance to chemical insecticides. Continued monitoring of resistance trends and mechanisms is essential for informing evidence-based resistance management strategies.

## Supplementary Information

**supplementary material 1. **Table S1 Description of mosquito larva collection sites for the WHO tube assay on Hainan Island, China.

**supplementary material 2. **Table S2 Primers used in this study.

**supplementary material 3. **Fig. S1 Examples of agarose gel electrophoresis banding patterns. Lanes 1–2: PCR products amplified using *Aedes albopictus* *kdr* primers; Lanes 3–4: PCR products amplified using *Culex quinquefasciatus* *kdr* primers; Lanes 5–6: PCR products amplified using *Anopheles* *kdr* primers; Lanes 7–12: PCR products amplified using mosquito *coxI* gene primers; Lanes 13–14: PCR products amplified using *Anopheles sinensis*-specific primers; Lanes “–”: negative controls; Lane M: DNA ladder marker.

**supplementary material 4. **Fig. S2 Pyrethroid resistance status of *Aedes aegypti* **a** and *Culex tritaeniorhynchus* **b** in Hainan.

## Data Availability

Nucleotide sequences generated in this study have been submitted to GenBank (Accession numbers PV533895-PV533904, PV764712-PV764746).

## Declarations

**Ethics approval and consent to participate**

Not applicable.
