# Supplementary figures and images for: Mechanisms of pyrethroid resistance in Culicidae mosquitoes from Hainan Island, China
Source: Parasit Vectors. 2025 Oct 14;18:411. doi: 10.1186/s13071-025-07052-y (PMC12522602; doi:10.1186/s13071-025-07052-y)

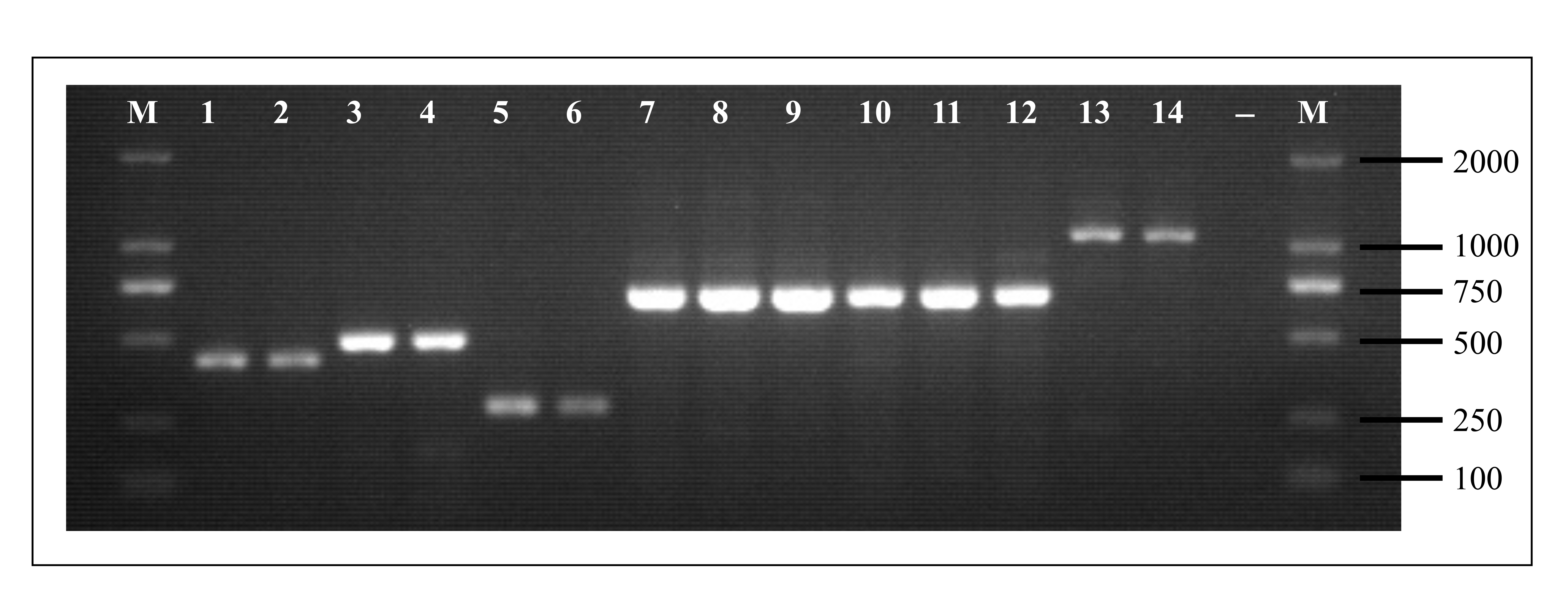

Supplement: Supplementary file 1 — supplementary material 1. Table S1 Description of mosquito larva collection sites for the WHO tube assay on Hainan Island, China. [file 13071_2025_7052_MOESM1_ESM.tif]

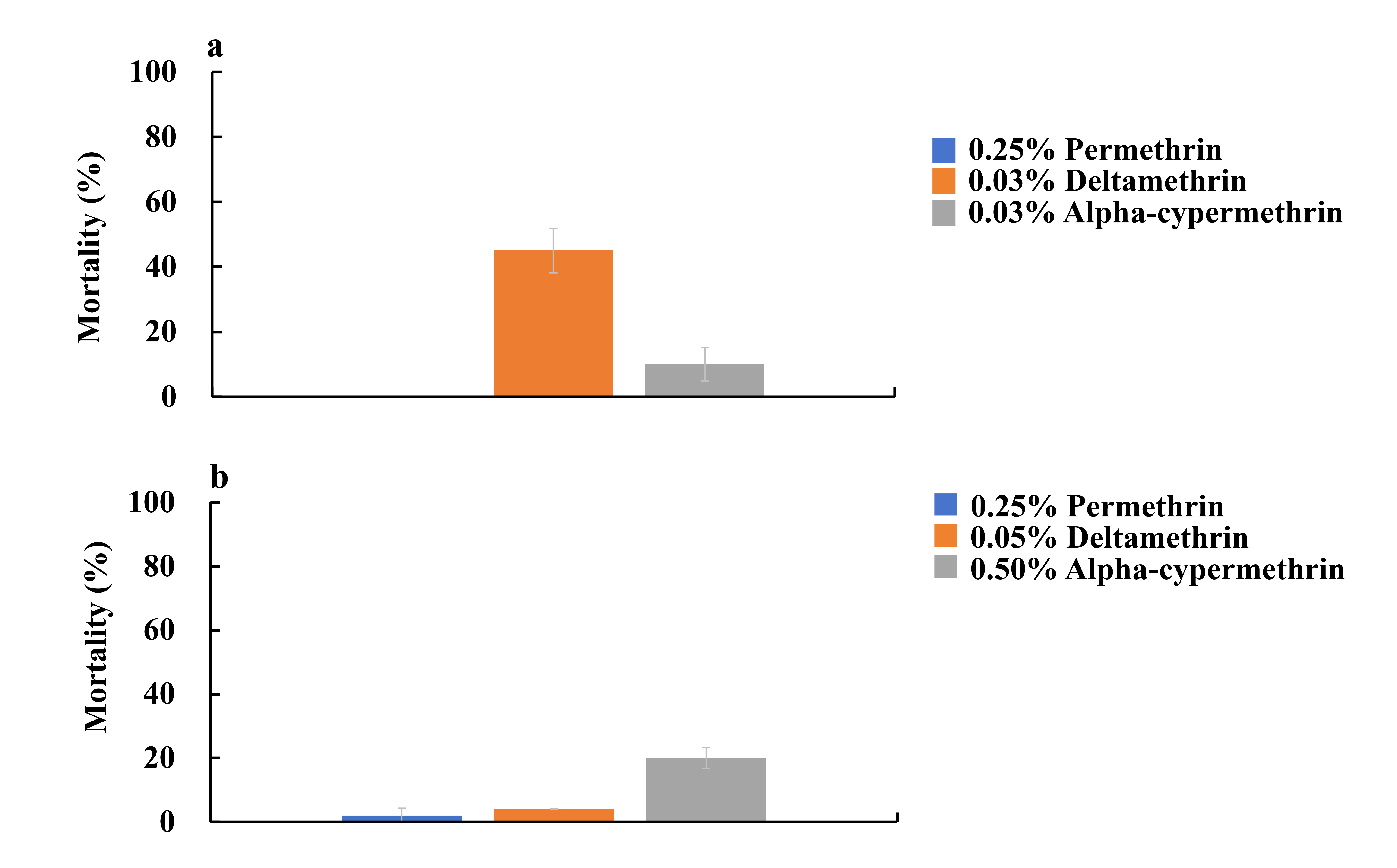

Supplement: Supplementary file 2 — supplementary material 2. Table S2 Primers used in this study. [file 13071_2025_7052_MOESM2_ESM.tif]
