# Supplementary material for: Mechanisms of pyrethroid resistance in Culicidae mosquitoes from Hainan Island, China
Source: Parasit Vectors. 2025 Oct 14;18:411. doi: 10.1186/s13071-025-07052-y (PMC12522602; doi:10.1186/s13071-025-07052-y)
Supplement: Supplementary file 3 — supplementary material 3. Fig. S1 Examples of agarose gel electrophoresis banding patterns. Lanes 1–2: PCR products amplified using Aedes albopictus kdr primers; Lanes 3–4: PCR products amplified using Culex quinquefasciatus kdr primers; Lanes 5–6: PCR products amplified using Anopheles kdr primers; Lanes 7–12: PCR products amplified using mosquito coxI gene primers; Lanes 13–14: PCR products amplified using Anopheles sinensis-specific primers; Lanes “–”: negative controls; Lane M: DNA ladder marker. [file 13071_2025_7052_MOESM3_ESM.docx]

| Table S1. Description of mosquito larvae collection sites for WHO tube assay in Hainan Island, China. | | | |
| --- | --- | --- | --- |
|  |  | Coordinates | |
| Mosquitoes | Study site | Latitude (N) | Longitude (E) |
| *Ae. albopictus* | Haikou | 20°01'12" | 110°26'18" |
|  | Qionghai | 19°14'56" | 110°29'58" |
|  | Wenchang | 19°59′10″ | 110°19′49″ |
|  | Danzhou | 19°37'60" | 109°11'56" |
|  | Tunchang | 19°20'02" | 109°59'48" |
|  | Baoting | 19°58′35″ | 110°20′02″ |
|  | Chengmai | 19°45'00" | 110°10'38" |
| *Ae. aegypti* | Changjiang | 19°59′03″ | 110°19′48″ |
| *Cx. quinquefasciatus* | Haikou | 19°58'38" | 110°20'39" |
|  | Qionghai | 19°04'04" | 110°22'12" |
|  | Wenchang | 19°59′10″ | 110°19′49″ |
|  | Danzhou | 19°37'60" | 109°11'56" |
|  | Chengmai | 19°44'59" | 110°11'02" |
| *Ar. subalbatus* | Haikou | 19°58'13" | 110°21'33" |
|  | Qionghai | 19°04'04" | 110°22'12" |
|  | Tunchang | 19°20'02" | 109°59'48" |
| *Cx. tritaeniorhynchus* | Qionghai | 19°14'56" | 110°29'58" |
| *Ae. albopictus*,  *Cx. quinquefasciatus* | Haikou | 19°58'38" | 110°20'39" |
|  | Qionghai | 19°04'04" | 110°22'12" |
|  | Danzhou | 19°37'60" | 109°11'56" |
|  | Chengmai | 19°31'55" | 110°05'10" |
|  | Lingao | 19°54'42" | 109°50'19" |
