# Supplementary material for: Mechanisms of pyrethroid resistance in Culicidae mosquitoes from Hainan Island, China
Source: Parasit Vectors. 2025 Oct 14;18:411. doi: 10.1186/s13071-025-07052-y (PMC12522602; doi:10.1186/s13071-025-07052-y)
Supplement: Supplementary file 4 — supplementary material 4. Fig. S2 Pyrethroid resistance status of Aedes aegypti a and Culex tritaeniorhynchus b in Hainan. [file 13071_2025_7052_MOESM4_ESM.docx]

**Table S2. Primers used in this study**

| Primer Name | Primer sequence (5'→3') | Annealing Temperature (℃) |
| --- | --- | --- |
| *An. sinensis* - F | TGTGAACTGCAGGACACATGAA | 52 |
| *An. sinensis* - R | AGGGTCAAGGCATACAGAAGGC |  |
| LCO1490 - F | GGTCAACAAATCATAAAGATATTGG | 55 |
| HC02198 - R | TAAACTTCAGGGTGACCAAAAAATCA |  |
| *Ae.albopictus - kdr* - F | GAGAACTCGCCGATGAACTT | 60 |
| *Ae.albopictus - kdr* - R | TAGCTTTCAGCGGCTTCTTC |  |
| *Culex* - *kdr* - F | GTGGAACTTCACCGACTTC | 58 |
| *Culex* - *kdr* - R | GCAAGGCTAAGAAAAGGTTAAG |  |
| *An* - *kdr* - F | GACCATGATCTGCCAAGATGGAAT | 55 |
| *An* - *kdr* - R | GAGGATGAACCGAAATTGGAC |  |
